# Building Irish families through surrogacy: medical and judicial issues for the advanced reproductive technologies

**DOI:** 10.1186/1742-4755-5-9

**Published:** 2008-11-04

**Authors:** Eric Scott Sills, Clifford M Healy

**Affiliations:** 1 Sims International Fertility Clinic, Dublin, Ireland; 2 Department of Law, University College Cork, Cork, Ireland

## Abstract

Surrogacy involves one woman (surrogate mother) carrying a child for another person/s (commissioning person/couple), based on a mutual agreement requiring the child to be handed over to the commissioning person/couple following birth. Reasons for seeking surrogacy include situations where a woman has non-functional or absent reproductive organs, or as a remedy for recurrent pregnancy loss. Additionally, surrogacy may find application in any medical context where pregnancy is contraindicated, or where a couple consisting of two males seek to become parents through oocyte donation. Gestational surrogacy is one of the main issues at the forefront of bioethics and the advanced reproductive technologies, representing an important challenge to medical law. This analysis reviews the history of surrogacy and clinical and legal issues pertaining to this branch of reproductive medicine. Interestingly, the Medical Council of Ireland does not acknowledge surrogacy in its current practice guidelines, nor is there specific legislation addressing surrogacy in Ireland at present. We therefore have developed a contract-based model for surrogacy in which, courts in Ireland may consider when confronted with a surrogacy dispute, and formulated a system to resolve any potential dispute arising from a surrogacy arrangement. While the 2005 report by the Commission on Assisted Human Reproduction (CAHR) is an expert opinion guiding the Oireachtas' development of specific legislation governing assisted human reproduction and surrogacy, our report represents independent scholarship on the contractual elements of surrogacy with particular focus on how Irish courts might decide on surrogacy matters in a modern day Ireland. This joint medico-legal collaborative also reviews the contract for services arrangement between the commissioning person/s and the surrogate, and the extent to which the contract may be enforced.

## Background

Surrogacy describes an alternate means of conception for individuals who are unable to conceive a child naturally. In surrogacy, one woman (surrogate mother) carries a child for another person/s (commissioning person/couple), based on an agreement before conception requiring the child to be handed over to the commissioning person/couple following birth.

The basic idea of surrogacy is an understandably private matter. Yet there is nothing new in the concept of surrogacy, which probably began with civilisation itself. While Biblical references include Sarah (Genesis 16:4) and Rachel (Genesis 30:3), perhaps less well-known allusions to surrogacy date from 1500 B.C. and are found among the artefacts of the Hurrians of Mesopotamia [1]. Certainly surrogacy was practiced in classic Greek society as early as the 4^th ^century B.C. [2], and in medieval Tuscany, one mother's daily correspondence preserved from around the year A.D.1500 provides another perspective of surrogacy [3]. The longest intact personal inscription that survives from ancient Rome (known as *Laudatio Turiae*–a late 1^st ^century B.C. epitaph) includes a tender reference to surrogacy as antiquity's common-sense remedy to childlessness [4].

Contemporary advances in medical science have taken the application of surrogacy to a new level of sophistication, and over the past two decades surrogacy has attracted legislative attention worldwide. In clinical practice, surrogacy includes arrangements whereby a woman agrees to become pregnant (either by artificial insemination or via embryo transfer), and carry a child to term with the intent to relinquish custody of that child upon its birth to the couple with whom she has made the agreement [5]. This agreement has the practical effect of causing a purposeful conception followed by voluntary surrender of the offspring by the birth mother, or shortly after birth [6].

A surrogate may have a purely altruistic reason for agreeing to carry a child for someone else, but generally this is done in exchange for compensation to the surrogate mother. Recent research has challenged the cultural assumption that "normal" women do not voluntarily become pregnant with the premeditated intent to surrender the child for money [7]. General public acceptance of this is increasing but remains somewhat limited [8]. Regarding the level of support for surrogacy, considerable variation exists across population sub-groups with some of this perception appearing to be influenced by prevailing media portrayals of surrogacy [9].

While generally favourable legislation relating to IVF had appeared in several jurisdictions by the end of the 1980's, this level of acceptance did not initially extend to surrogacy. For example, South Africa, England and Australia had produced essentially negative legislation on surrogacy by 1990 [10]. Even though the United Kingdom's *Surrogacy Arrangements Act 1985 *(SAA 1985) [11] prohibits payments for surrogacy arrangements, financial compensation and reimbursement of reasonable expenses to the surrogate are considered lawful. This contrasts with the United States model where (depending on the State) a surrogate may be paid for the service of carrying the child to term. The main concern with the SAA is that, even where a surrogate has agreed to carry to term a child which comprises of the gametes of the commissioning couple, under the latter Act, the surrogate will be regarded as the legal parent of the offspring, even though she holds no genetic tie with the child.

Although distinct types of surrogacy exist and the definitions of each have occasionally resulted in confusion, the status of the surrogate herself (the woman carrying the child) determines the type of surrogacy. Does the surrogate have a direct genetic link to the child? How this question is answered establishes the classification of each surrogacy arrangement:

1. *Partial Surrogacy (traditional surrogacy)*: This is currently the most common form of surrogacy arrangement, where the carrying female is fertilised with the commissioning male's sperm (either by sexual intercourse or assisted insemination). Because this type of surrogacy does not rely on the advanced reproductive technologies, partial surrogacy may be undertaken privately without the aid of a medical practitioner.

2. *Full Surrogacy (gestational surrogacy): *This relies on in vitro fertilisation (IVF) and therefore involves minor surgery and the expertise of highly trained medical staff. The commissioning couple may provide both sperm and ovum, while the surrogate provides the uterus where the resulting embryo created by IVF is transferred.

In the latter arrangement, the resulting child shares the same genetic composition as the commissioning couple, providing that both the sperm and ovum of the couple were used, whereas in the former arrangement, the child is genetically related to both the commissioning father and the surrogate mother (as the oocyte source). When a dispute arises, the genetic makeup of the child usually carries judicial weight in deciding where a child is more suitably placed. A typical surrogacy contract would stipulate the objective of the contract including the intention of the commissioning person(s) to be the legal parents of the child. The surrogate would merely be providing a service which is necessary in order for the contract to be carried out. Strict application of the principles of contract law would conclude that a child born through gestational surrogacy, thereby exclusively comprising the genetic makeup of the commissioning couple (or partly, where the sperm or ovum of one of the parties was donated), should never be legally classified as offspring of the surrogate (as appears to be the case in some countries) simply because the surrogate delivered the infant.

Similarly, with partial surrogacy the courts typically interfere only when confronted with a custody dispute and in particular, when required to determine the best interests of the child. It may be claimed that both the commissioning couple and the surrogate mother would have an equal legal right to claim custody of the child in this context. However, as noted above with full/gestational surrogacy, the surrogate has no genetic connection to the offspring and therefore the commissioning person(s) should have a stronger claim to be awarded custody. Thus, depending on the particulars of the agreement between the parties, the courts, as will be seen throughout this paper, should reserve judgement until all the facts are delicately assessed with reference to the following; the intention of the parties, the genetic parent/s of the child and most importantly, the best interests of the child.

### Current Legal Position in Ireland

At present, Ireland has no legislation governing surrogacy arrangements or any forms of assisted reproductive medicine. The *Commission on Assisted Human Reproduction *(CAHR), chaired by Dr. Deirdre Madden of University College Cork, concluded in 2005 as the government's first attempt to address the need for statutory regulation in the area of assisted human reproduction [12]. Overall the CAHR report was widely accepted as being a positive step parallel to other EU jurisdictions. The portion of the Commission's report specifically dealing with surrogacy may be seen as a reflection of current society's social, moral, and ethical milieu. The report recommended that there should be *"no prohibitions regarding marital status, gender or sexual orientation of the commissioning person(s)"*, and as such, is generally viewed as taking a progressive step towards a socially diverse Ireland.

Although the Oireachtas Health Committee (OHC) has assessed the findings of the CAHR report, thus far, no Parliamentary action has resulted from the work of the OHC. Until the Oireachtas passes a law specifically addressing surrogacy, Ireland will remain a blank slate leaving it to the judiciary to determine what rights either party in a surrogacy arrangement may have. In the absence of relevant legislation here, women in Ireland have very limited access to surrogacy and only recently have pregnancies from surrogacy here been described [13]. Estimates of the number of Irish couples participating in surrogacy arrangements outside of Ireland are difficult to calculate, however a voluntary organisation based in the United Kingdom, Childlessness Overcome Through Surrogacy (COTS), has records of its agency assisting 10 Irish couples to become parents to 15 children over the years with no disputes arising from such arrangements (*personal communication*).

In an Irish context, if a surrogacy arrangement were to require adjudication now, it is likely that judges would look to the Medical Council of Ireland (IMC) as the statutory body empowered to regulate the practice of medicine at a national level. One of the functions of the IMC is to provide guidance on professional ethics to physicians, and the Council publishes its "Guide to Ethical Conduct & Behaviour" every five years for this purpose. However, the IMC has not been progressive about regulating the advanced reproductive technologies. For example, a provision restricting IVF only to married couples was retained until 1994, and the most recent edition of the IMC ethical "Guide" (2004) did not even mention surrogacy [14].

The Irish judiciary also could turn to precepts of common law supplemented by the 1937 Irish Constitution [15]. The closest statute that might reasonably be applied in this setting would approximate the same set of facts pertaining to the adoption of a child by the commissioning couple, and would therefore be conditional upon the surrogate mother relinquishing custody after approval by the Adoption Board, under the *Adoption Acts 1952–1998 *[16]. This hypothesis leads to the issue of whether the *Adoption Acts 1952–1998 *in their present state, would fit properly as part of a solution to disputed surrogacy arrangements. At least two factors must be addressed for this analysis.

First, the Adoption Acts stipulate that a mother cannot lawfully relinquish her parental rights by offering a child for adoption *before *its birth, even if she wishes to do so. The CAHR's dissenting opinion position paper on surrogacy focused strongly on this key distinction, and speculated that this aspect of the Adoption Acts was specifically crafted with a view to keep mothers from recklessly waiving a constitutional right, until it was absolutely clear that they had a full understanding of what they were, in fact, giving up [17]. An effort to resolve a surrogacy dispute by applying the *Adoption Acts 1952–1998 *is therefore problematic, because all surrogacy contracts depend on the parties' mutual agreement well in advance of conception.

The second issue deals with the Adoption Acts' treatment of payment to the birth parents. In surrogacy arrangements, some form of monetary compensation would almost invariably pass from the commissioning person/s to the surrogate (birth mother). This may be so even in the context of a genuinely altruistic arrangement, where the commissioning person/s offset the costs of the delivery procedure, and perhaps clinic expenses associated with prenatal care. Although such expenses may only cover the above procedures, this trace level of economic involvement may be regarded as a contravention of the Adoption Acts.

When this matter was investigated in 2007, the Irish Adoption Board indicated that it receives at least two applications per year from commissioning couples seeking to adopt a child born through surrogacy. The Board has permitted the commissioning person/s to cover the general expenses of the surrogate so long as this arrangement is not coercive or driven by profit. This is so, even though Section 42 of the Principal Act clearly states that any payment or other reward will be in direct contravention of the Act. However, one must bare in mind that the principal Act was penned in 1952 and did not foresee the practical realities and difficulties in conceiving – nor did it foresee the plethora of contemporary advances in medical science.

The practical effect of this (permitting the adoption by the Board), in an Irish context, means that similar payments (provided they are reasonable) flowing from the commissioning person/s to the surrogate are very unlikely to be viewed as a contravention of the Adoption Act. However, when evaluating surrogacy disputes, the courts need to contemplate a number of key factors including the biological (genetic) status of the child, the contractual intention of the parties, and most importantly, the best interests of the child. The matter of '*reasonable expenses*' should also be assessed to the extent that this is a necessary element of the surrogacy arrangement, yet assuming the above factors were properly considered, the issue of expenses would not hinder the true essence of the contract and would become only of secondary importance.

### Surrogacy Models in Foreign Jurisdictions

Given the absence of relevant Irish legislation and case law specific to surrogacy, it is useful to explore jurisdictional strategies adopted by persuasive authorities elsewhere. Although there are several countries that have, in either a legislative or judicial capacity, established legal precedent on issues pertaining to surrogacy, two (*i.e*., United Kingdom and California) are studied here with a view to compare and contrast these approaches.

#### United Kingdom

In the United Kingdom, the legislation pertaining to surrogacy is the SAA 1985 [11]. This legislation came in response to a highly publicised surrogacy case known as *'Baby Cotton' *[18]. This case arose prior to the introduction of the SAA and involved a partial surrogacy arrangement that had been set up through a commercial agency in the United States. The surrogate agreed to be inseminated with the commissioning fathers' sperm and, upon the birth of the child resulting from this procedure, was content to relinquish custody. When a custody dispute arose, the local court intervened and Baby Cotton was temporarily made a ward of court. Mr. Justice Latey found the commissioning couple to be suitable parents and ultimately ruled that the sole care and custody of the child should be awarded to them, and that they were therefore permitted to take the child out of the United Kingdom.

The first case addressing a disputed surrogacy arrangement in the United Kingdom was actually decided in 1978, although it was not reported until 1985 [19] when the controversial *Baby Cotton *case came before the courts. In that first judicial exploration of contested parentage arising from surrogacy, the court held that "the agreement was void on the grounds of public policy", but subsequently gave the child's biological father visitation rights.

The Baby Cotton story attracted considerable media attention and was subsequently drawn into Parliamentary debates, which began to question the *a priori *moral acceptability of surrogacy itself. Against this background, the SSA 1985 was passed which prohibited the commercialisation of surrogacy by third-parties or agencies, but did not prevent the act of surrogacy itself. Since its enactment, the Act has received criticism on the basis that it was enacted due to "moral panic" [20] and was described as a "stopgap measure driving surrogacy underground" [21].

While the Act was created with the laudable goal of combating the commercialisation of surrogacy in the United Kingdom, it was unclear how the exchange of money would be "less commercial" if given directly to the surrogate mother by the commissioning couple, instead of by an agency (ostensibly acting on behalf of the commissioning person/s). The SAA 1985 therefore aimed to discourage surrogacy arrangements by prohibiting intermediaries, but in practical terms it had the effect of proliferating amateur surrogacy agreements by outlawing professional, experienced help. It may be argued that legal advice and formal counselling for the surrogate mother has value, as it would reduce the risk of separation issues that may be encountered when she delivers the child, compared to the circumstance where the surrogate mother would not have access to such resources. The involvement of a regulated agency providing this professional service (for a fee defrayed by the commissioning person/s), may thus be seen as contributing a highly technical yet beneficial service to inform all parties as they contemplate entering a surrogacy arrangement.

Under English law, the woman who physically gives birth to the child is currently classified as the legal mother irrespective of whether she is the genetic mother of the child. This means that in the United Kingdom the commissioning mother, who may in fact be the genetic mother of the child, will never automatically acquire legal parental responsibility for a child born through a surrogacy arrangement [22]. It should be noted that the SSA 1985 prohibits any agency to facilitate commercial surrogacy arrangements and prohibits advertisements for surrogates or to recruit a surrogate in the United Kingdom. In contrast, the *Human Fertilisation and Embryology Act 1990 *(HFEA 1990) [23] confirms the common law position that such contracts are unenforceable in the United Kingdom. HFEA 1990 does not specifically address surrogacy arrangements, but S.30 does provide measures the commissioning couple may take in applying for adoption or guardianship rights.

From this analysis, it may be concluded that English law does not presently prohibit surrogacy arrangements but does criminalise them if such agreements include a commercial element. Any contracts made of a commercial nature, beyond what may be deemed as covering reasonable expenses will be declared void in accordance with English law [24]. The legal approach to surrogacy in the United Kingdom has also been criticised because it regards as irrelevant the intention of the parties involved. This is perhaps best illustrated in sec. 27 of HFEA 1990 which states that a surrogate is defined as the legal mother of the child born, even though she may be genetically unrelated to the child and have no wish to be held as its legal mother.

#### State of California (USA)

In the 1993 case *Johnson v. Calvert *[25], the California Supreme Court extended existing California Family Law statutes to protect all parties in surrogacy arrangements and in oocyte donation pregnancies. *Johnson v. Calvert *presented a straightforward example of a dispute arising from a gestational surrogacy agreement. The commissioning woman, Crispina Calvert, had undergone a hysterectomy and was therefore unable to carry a child but still had ovaries and was therefore able to produce her own eggs. She and her husband entered into an agreement with a surrogate, Anna Johnson, to be paid a fixed fee in return for the bringing to term the commissioning couple's child created from their gametes. When the relationship between the parties deteriorated, both parties applied to the court for an order of parentage. The surrogate (carrier), Ms. Johnson, claimed that she was being exploited due to the fact that she was a woman of lower economic status. The court disagreed and stated that considering the contract there was no coercion or duress involved, and that "there had been no proof that surrogacy contracts exploit poor women...". The California State Supreme Court affirmed the lower Court ruling that a gestational surrogacy contract was legal and binding.

The California Court further reasoned that there were two distinct ways to prove maternity using an existing statute (*i.e*., California Family Law Code). The first method was by proof of giving physical birth to the child, and the second method was by proof of genetic consanguinity (blood tests). But since *Johnson v. Calvert *involved twodifferent women who could each fulfil different parts of the statutory requirements, the Court held that the woman who intended to "bring about the birth of a child that she intended to raise as her own – is the natural mother under California law."

An earlier analysis of this case [26] concluded that the California Court appreciated the complexities of IVF and the level of planning and coordination needed to achieve a pregnancy, and regarded the *intellectual conception *of the child as being the fundamental cause of the child's creation, "But for the commissioning parents setting out to find a surrogate to carry their embryo, this child would have never come into existence." In other words, if the commissioning couple takes the necessary logistical steps to identify and engage a surrogate mother with the intention of the surrogate carrying to term a child that is genetically related to one (or both) of the parties, and the surrogate voluntarily agrees to such terms, then that contract should in all instances be given validity in favour of the commissioning couple based on that intent.

This important California decision is in contrast to some other jurisdictions in the Unites States where absolute prohibition on surrogacy has been effected by completely banning the practice. The New Jersey Supreme Court decided a well-known American surrogacy case, *In the Matter of Baby M *[27], holding that the agreement itself was void but nevertheless awarded custody to the commissioning couple. California law has paved the way for prospective parents, surrogates, and egg donors to be reasonably certain that their *intentions*, as expressed by their formal surrogacy agreement, will be respected. California courts have consistently upheld the intended parents' rights and obligations of parenthood when they use a surrogate or egg donor to help create a family. This result will generally hold true regardless of whether the parents use their own genetic material, donated eggs, or artificially inseminate a surrogate.

The California approach is acknowledged as completely opposite to that operating in the United Kingdom. In California, a contractual model is viewed as giving rise to the rights and obligations of the joint intention of the parties to the contract. Under a contract for services, consideration is given by way of placing an embryo or commissioning person's sperm into the surrogate. Prior to making such an agreement, both parties are fully aware of the basic terms of the contract; specifically, that the surrogate will carry the child to term with the intention that she will, upon its birth, give that child over to the commissioning couple.

### Surrogacy Contracts

A surrogacy contract is a contract no different to any other contract as it essentially relates to the agreement or promise made by both parties: contract law is primarily concerned with agreements that involve one party, or each party, giving an undertaking or promise to the other party [28]. The rights and duties of the surrogate stem from two basic promises that she makes to the commissioning couple. First, she promises to be treated with the commissioning couple's genetic material (partial/full surrogacy) and carry the child to term. The surrogate will also give an assurance that she will attend regular prenatal appointments so as to ensure the health and safety of the foetus.

Secondly, the surrogate will promise to surrender all rights in the child to the commissioning couple. This latter promise may become complicated if the surrogate is married, as the law presumes that a child born to a married woman is the child of the woman and her husband. However, this presumption is rebuttable and thus, the commissioning couple should from the outset, make it a term of the contract that the surrogate and her husband explicitly agree to make no claim to the resulting child; without this statement, the intention of the parties may be undercut. Such a provision would help reduce emotional strain and the probability of litigation, and would avoid harming the child by involving it in custody proceedings [29].

A surrogacy arrangement based on contractual intention should not be designed to commodify offspring. Surrogacy arrangements do not deal with fungibles and must not encourage a system where children are treated as goods that may be contracted in and out of. While the notion of surrogacy could understandably figure centrally in the arena of Irish family law, when examining the matrix of relationships embraced by surrogacy, one may see that surrogacy also has a basis in contract law. As with all contracts, they are designed to protect the interests of both parties as well as to bring to fruition, the express and implied terms of the contract. This perspective derives from the basic agreement made between the surrogate and the commissioning couple; the surrogate agrees to carry the foetus to term, for the benefit of the commissioning person/s and, the latter agree to re-compensate the surrogate for her time and expense in carrying out said procedure, of which, would not be possible without her agreement.

A proposed contractual model for surrogacy in Ireland could effectively be based on the common law principles of freedom to contract, as enunciated in *Johnson v. Calvert *[25]. From a contract law standpoint, Irish courts could assess several factors for consideration when adjudicating a surrogacy dispute. Assuming all elements of a valid contract exist, *offer and acceptance *of carrying out the surrogacy arrangement and thereby electively agreeing to all terms of the contract, *consideration *would be regarded as given by consenting to have the embryo/sperm transferred to the surrogate's uterus, and finally, the contract would on the birth and relinquishing of the child, be classified as having satisfied the *intention *of the parties concerned and thereby completed. It should be noted that whether the particular terms of the contract are "reasonable" is not technically at issue. Indeed a surrogacy contract could consist of any number of terms/clauses, yet it may be assumed that all such terms are reasonable because they were freely acceded to by all parties involved [30].

### Intent of the parties

"*Intention*" in a surrogacy agreement connotes the same meaning as in other legal contracts. Courts look to what the parties intended by examining the contract in a prima facie fashion. The contract itself should detail the provision of a service to and on behalf of the commissioning couple (the intended parents), and the contract language would stipulate that the fundamental purpose of the agreement is to provide the intended parents with a child which is genetically theirs. In consideration for the contractual promise, the surrogate (or host) would be recompensed for her expenses incurred during pregnancy. Whether or not this compensation might conflict with Ireland's Adoption Act, as presently configured, cannot be known with certainty. However, as mentioned above, provided such payments are viewed by the Board as "reasonable" between the parties, the courts may infer that they do not contravene the Acts.

Recognising the aforementioned distinctions in other legal systems, it can be noted that courts in the United Kingdom have ruled that the best possible approach in relation to the issue of fees was to 'disregard the morality or immorality of the arrangement itself...' and to decide on the best interests of the child [18,31]. Furthermore, California case law has further shown that courts will award legal parentage to the intended parents of the child irrespective of the gestational arrangements.

This was precisely demonstrated in *Re Marriage of Buzzanca *[32], where the court considered a more complicated IVF case involving an egg donor, extending the opinions from *Johnson v. Calvert*. In this conflict regarding donor oocytes and gestational surrogacy, the Court held that where a child is born as a result of a surrogacy arrangement to a couple who are not the biological (genetic) parents, the intention of that couple to become parents would still be sufficient to render them the legal parents of the child. The Court reasoned in *Buzzanca *that the intended mother to a surrogacy contract using a donated egg could prove she was the mother under existing *California Family Code Section 7610 *by virtue of her consent to undergo "...a medical procedure which results in a pregnancy and eventual birth of a child" [32].

When given an opportunity to decide on such complex cases involving surrogacy disputes, Irish courts in assessing legal parentage and the best interests of the child, might seek some guidance from the legislative and judicial model currently used in the United Kingdom. However, as mentioned at the introductory stage, the SAA in the United Kingdom will only provide assistance to the commissioning couple where the surrogate willingly relinquishes the baby and permits the adoption. Thus, the California model would offer wider protection to the true and intended parents of the infant, taking into account the best interests of the infant at all times. Alternatively, the Irish judicial system could assess the contractual intentions of all parties concerned, or as the court opined in the *Calvert *decision, the '*intellectual conception*' of the child and the '*but for*' test adopted by other jurisdictions. These considerations should facilitate the Irish courts' interpretation of the terms of the agreement, the status of parentage in light of advances in modern medicine, and most importantly, the best interests of the child.

### The Constitution and contract law in Ireland: Conflicting philosophies?

Moral philosophers have often debated the topic of obligation in relation to keeping one's promise. It is believed that where one simply agrees to keep a particular promise, they should be held up to that promise as moral obligations are created by an individual saying so. Accordingly, legal philosophers are often influenced by moral philosophical theories about 'promising' when exploring contract-based agreements. This formulation may become somewhat complicated when viewed in the framework of the Irish Constitution, however.

Can it be said that the contract model combined with the Irish Constitution advances a couple's basic right to procreate and establish a family? The 1937 Constitution of the Republic of Ireland [15] explicitly acknowledges the special status of the family as *'the natural primary and fundamental unit group in society'*, and as such recognises the natural rights of its members. However, the well-intentioned protections afforded to the Irish family by the Constitution may be variously interpreted as either supporting or proscribing the practice of surrogacy. For example, it may be argued that the commissioning couple is pursuing a family and the only way this goal can possibly be achieved is through surrogacy. The couple might further contend that if surrogacy contracts were deemed invalid, unconstitutional, and/or unenforceable, the lack of a legally meaningful surrogacy mechanism in Ireland would effectively deprive them of the opportunity to have a family–a family which in turn, is ostensibly protected by the Constitution itself.

When deciding a case involving full (gestational) surrogacy, an Irish court would hear compelling arguments in favour of the commissioning person/s since in this setting the child would share the same genetic composition as the commissioning couple, and have nothing in common genetically with the surrogate. Other jurisdictions have acknowledged that in disputed gestational surrogacy arrangements, both women involved could be considered the child's natural mothers. However, when intention and genetics informed their deliberations, the California Court regarded the legal mother as the genetic mother, and as such, custody was awarded accordingly. Considering the family rights guaranteed within the Irish Constitution, it could be argued that this gives more emphasis on the commissioning couple as opposed to the surrogate (birth mother). Therefore, in order to safeguard that familial relationship, the Irish courts could recognise a contract-based model in the absence of specific surrogacy legislation.

### Public policy developments

In response to the widespread availability of the advanced reproductive technologies, many jurisdictions have developed regulations and/or legislation designed to anticipate problems or disputes arising from this technology.

#### Ireland

The majority of the CAHR were of the opinion that surrogacy should be regulated by a separate licensing body specifically for surrogacy, and also recommended the remit of the Adoption Board be extended to include surrogacy [12]. In assessing the contractual issues in a surrogacy arrangement, the Commission stated that due to the various forms of surrogacy arrangements available including contractual, commercial, altruistic, anonymous and intra-familial and genetic surrogacy, "the rules that may be envisaged for one type may not necessarily fit the others" [12].

The Commission recommended that for traditional (partial) surrogacy, Irish courts would classify the surrogate as the legal mother due to genetic and gestational reasons. The Commission did consider the legal position in gestational (full) surrogacy, where the surrogate carries an embryo that is not genetically her own, and stated, "both genetics and gestation play a necessary and equally important role in bringing the child into existence". Furthermore, the Commission discussed legal parentage in surrogacy arrangements as follows: "the rights based on the 'intent of reproduction', in other words what all parties intended from the outset of the arrangement, should form the basis of recommendations on legal parentage in cases of surrogacy." [12]

After considering several options related to the legal parentage of a child born through surrogacy, the majority of the panel recommended that the child born through surrogacy should be '*presumed*' to be that of the commissioning couple [12]. This position by the CAHR can direct the Oireachtas in developing legislation to grant parental and legal status in gestational surrogacy arrangements. Thus far, the Government has not commented on the Commission's recommendations.

#### United Kingdom

It has been more than 20 years since the government of the United Kingdom produced a formal enquiry into *Human Fertilisation and Embryology*, the resulting document known as the Warnock Report [33]. This was followed by a related regulatory effort, the Brazier Report [34]. At the time of its publishing, the Warnock Report assented to most forms of reproductive technology such as IVF, artificial insemination and egg donation. Curiously, the majority voice in the Warnock Report took a rather negative view of surrogacy and predicted that it would "disappear" or "wither on the vine." Moreover, the Warnock Report considered surrogacy to be "inconsistent with human dignity that a woman should use her uterus for financial profit and treat it as an incubator for someone else's child." But neither the SAA 1985 nor HFEA 1990 fully embodied the recommendations of the Warnock Report.

The Brazier Report [34] reviewed surrogacy arrangements to ensure that "the law continued to meet public concerns." Brazier recognised that since the time of the SAA 1985 and the findings of the Warnock report, public opinion had changed on surrogacy, and indeed that surrogacy had become an "acceptable alternative" to other forms of reproductive treatments. Brazier recommended that in order to consolidate and reform the law, a new Surrogacy Act was needed [35]. The Brazier report contended that "legal considerations" of surrogacy led to the conclusion that "any financial arrangements" beyond reasonable expenses "has to be regarded as a form of child purchase" and thus, Brazier recommended that surrogacy contracts containing such provisions continue to be unenforceable. However, the Brazier Report advocated allowing surrogacy to continue albeit in a regulated fashion, and with the ban on commercialisation to remain in place [34].

The Brazier Report concluded that compensation between consensual adults for the service of bringing to term the commissioning person/s child, should not be regarded as an act of purchasing children but rather payment for services rendered. In most surrogacy relationships, at least one or both commissioning persons are in fact genetically related to the child born from the surrogacy arrangement. Wilson has said that where one or both parents seek to adopt a child born through surrogacy, the *Adoption Act 1952 *may permit this because the arrangement is considered a form of 'step-family adoption' (where either one or both persons are biologically related to the child). According to this view, the surrogate mother effectively is seen as 'surrendering or abandoning' her right to custody of the child. Thus, payment of reasonable expenses is considered reimbursement to the surrogate for loss of earnings and any medical procedures carried out as a result of her services [16].

### Surrogacy contracts and Irish public policy

All contracts contain similar elements; whether the document concerns completing renovations to one's business or home, or agreeing to carry a child to term for someone else. All contracts should include the structure necessary to deem it valid: offer, acceptance, and consideration. Even when a contract has these required components, issues of moral consideration may complicate the question of whether courts would hold surrogacy contracts valid [36]. This is because all legal systems reserve the right to declare a contract void if it is legally or morally offensive, contrary to public policy, or if it involves the commission of an unlawful act [28].

#### Grounds for invalidation

A surrogacy contract may be rendered invalid when its terms describing compensation for "expenses" are critically viewed as exploitative, thus creating an impression of commercialisation. Expenses have come to include monetary payment to the surrogate for her medical treatment, her loss of earnings associated with pregnancy and other general expenses (*i.e*., maternity clothes and travel) calculated at a standard consistent with current market trends. In Ireland, this exchange of expense payments from the commissioning person/s to the surrogate could be contentious because the *Adoption Act 1952 *specifically prohibits a birth parent from receiving or agreeing to receive 'any payment or other reward in consideration of the adoption of the child'. It therefore forbids any person from giving or agreeing to give any such payment or reward. Courts in Ireland could take the view, irrespective of whatever label is put on the agreement, that "the reality of the contract is that it is an agreement to pay a fee in connection with an adoption", and therefore illegal [37].

English judges have tended to permit expense payments to the surrogate, provided they are reasonable, regarding such compensation as somewhat incidental to the agreement itself. This evokes a troublesome question: If a surrogacy case were to come before Irish courts today in the absence of surrogacy legislation, would the adoption of one's own genetic child be in contravention to the *Adoption Act 1952*? Upon a strict reading of the Section 42, the answer would be yes. However, as noted above, the Adoption Board will not classify reasonable expenses (medical or other) as contravening the Acts. One reason this agency has facilitated such adoptions is because one or both of the parents seeking the adoption is legally and genetically the parent/s of the child they seek to adopt (*personal communication)*.

#### Basis of illegality

The prevailing historical view was that any act of surrogacy diminished women and was degrading to all females. One of the central challenges confronting surrogacy is whether it is morally or ethically just, and whether in view of such concerns, the courts must assess the enforceability of surrogacy contracts. As such, the courts when looking at matters of public policy take account of the 'common good' and the changing perceptions of society as the validity of such contracts is evaluated.

Judges have never enforced contracts which, in the interests of morality and the common good, are regarded as contrary to public policy. In a French surrogacy case brought before the *Cour de Cassation*, the court held that surrogate motherhood must be regarded as lawful and "not contrary to public policy" and the "adoption is in accordance with the interests of the child" [38]. Although courts are quick to strike down illegal or morally offensive contracts, what may once have been regarded as morally doubtful may over time become socially and ethically acceptable. Modern reproductive technology has given many individuals an opportunity to create a family through various medical and surgical procedures. In light of these developments, it is unlikely that surrogacy would be viewed as objectionable or morally offensive by Irish courts. Indeed, it could be a delicate matter for a court to disparage and invalidate a surrogacy contract without to some degree also entangling the offspring who would be at the very centre of the dispute. By doing so, the resulting child would almost certainly be tainted with judicial disgust, an outcome antagonistic to "the best interests of the child" doctrine.

Although the courts of each jurisdiction seek to promote and protect the best interests of the child, this outcome is not always evident. An example of this may be found in the first surrogacy dispute litigated in Australia, *Re Evelyn *[39]. The outcome of this 1998 decision resulted in a highly disruptive environment for all parties concerned. While at the time, Australia had a complete prohibition on surrogacy, the court did not take this into account but decided instead to regard with more gravity the maturity of the parties in what was described as an altruistic, well-intentioned arrangement. However, the judge here decided that even though Evelyn had spent two years with her intended family, she should be returned to her birth mother (surrogate) to be raised. The intended parents were given shared long-term responsibility. It is anticipated that a ruling of this type would have limited influence on future Irish jurisprudence, as courts in Ireland tend to rely heavily on forensic or scientific literature which is in near universal agreement that the effect of such "parent switching" entails substantial psychological trauma to the child if bonds of attachment are formed and broken with the adoptive/intended parents [40,41].

### Breach of Contract & The Role of the Judiciary

Where a custody dispute arises between the surrogate and the commissioning person/s, what approach should the Irish courts take in resolving the dispute? It is our opinion that any future surrogacy dispute in Ireland ideally should be adjudicated on the basis of a *Three Point Test *to measure the critical aspects of the arrangement: *1) the genetic make-up of the child*, *2) the contractual intentions stipulated by the parties to the agreement*, and most importantly, *3) the best interests of the child*.

A genotypic approach would be fully consistent with the proposals of the CAHR report [12], which would validate that the gametes of one or both of the commissioning person/s were in fact used to bring about the creation of that child. Additionally, it would be important for Irish courts to consider the *intention of the parties *at the time of the agreement. Any decision may be supplemented by looking to the 'intellectual conception' of the child and the 'but for' test (*i.e*., 'but for' the intention of the intended parents, the child would have never existed).

Finally, we believe that the best interests of the child should outrank the former criteria in assessing legal parentage/custody in all cases. This last element of the equation, admittedly subjective, gives appropriate deference to the bench in quantifying the deciding factor in the outcome of the case. If, based on all available evidence, an Irish court determined that the birth mother (surrogate) rather than the commissioning couple best served the needs and interests of the child, then the previous two elements above would fail on such findings. However, if the court were satisfied that in a traditional surrogacy arrangement, both the birth mother and commissioning couple equally were eligible for custody, then the courts decision should rest on the first two factors in the *Test*.

#### Remedies

There are a number of ways both parties might breach a surrogacy contract. For example, the commissioning couple could decide not to pay for the medical expenses of the surrogate. Or, the surrogate might in some way intentionally harm the foetus by smoking or drinking during pregnancy. While these issues do not easily fall within the scope of our analysis, the commissioning couple (in the first scenario) may be held to be in breach of the terms of the contract and thus, compelled to uphold (specific performance) the terms of the contract. Similarly, in the second scenario the surrogate would be in breach of the express terms of the contract since it would be imperative for the commissioning couple to choose a healthy host to carry their child to full term. Accordingly, the surrogate may be liable to damages for emotional distress caused to the intended parents. Regarding situations where the surrogate refuses to relinquish custody of the child, what relief is available to the commissioning person/s? Are contractual remedies suitable in the context of surrogacy arrangements and how may such remedies be quantified?

#### Damages

Would an award of damages be an acceptable remedy in a surrogacy dispute? If so, how would the courts calculate this bearing in mind they would be assessing such damages in relation to (or, in exchange for) the loss of one's child? If legal parental custody of a child were to be granted to the birth mother (surrogate), would it be morally just to order restitution to the commissioning couple for the financial loss they had incurred, by directing reimbursement to them for *'expenses' *and medical treatments that they had paid to the surrogate (or her caregivers) during the gestational period? If the court simply reimbursed the commissioning couple for expenses, would this be a favourable remedy? A specific set of facts could lead an Irish court to decide that due to the child being partly that of the surrogate (genetically), that the commissioning person/s be required to pay for whatever expenses and costs associated with the child's upbringing, but with parental custody being assigned to the surrogate.

This scenario strongly favours the surrogate, and the commissioning person/s would find themselves in a situation with considerably less than they started with, as they would be forced to pay for offspring that they intended for themselves but would be legally denied full access to. This remedy appears unbalanced in that the surrogate would ultimately receive two benefits compared to none for the commissioning person/s, since the birth mother (surrogate) would enjoy the child as well as some form of guaranteed payment for its support. It is our belief that the Irish courts should decide the issue of damages in light of the party of whom it awards custody to:

• If the commissioning couple breach the terms of the contract then the surrogate should only be awarded compensation as per the terms of the contract, i.e., to put her in the position she would have been in had the contract been carried out fully. This would be in line with the recommendations of the CAHR report and the Adoption Acts 1952–1998.

• If the Surrogate breaches the contract (by refusing to relinquish custody), the courts should award the commissioning couple joint custody and a refund of all expenses that were incurred (medical and other).

#### Specific Performance

The application of specific performance would generally block the previous outcome by simply enforcing the agreement in place between the signatories to the agreement. The surrogate would, by court order, be forced to comply with the full intentions and objectives of the contract as stipulated therein, and therefore be obligated to give the child over to the commissioning parents. This judicial requirement of removing a baby from the bosom of its birth mother may first appear somewhat callous and indelicate, since the intrusion of legal manoeuvres near a moment traditionally given great family, social, and spiritual significance is unsettling. But the background and circumstances of such a birth would be quite different here. Indeed, the highly specialised context of the surrogacy arrangement is itself unnatural, and the ultimate conclusion of the contract would have specifically called for the bonds of motherhood to be reassigned to the commissioning person/s as *the *essential condition of fulfilling the agreement. Proper and total execution of the agreement occurs only when the surrogate specifically performs the tasks required of her, namely, by providing the commissioning person/s with the child that they themselves had conceptualised.

It must be admitted that for any future case in Ireland involving this emotional alloy of contract, family, and constitutional law, absolutes are difficult when determining the sensitive matter of parentage and child custody. Yet by utilising the *Three Point Test *as presented previously, Irish courts could (in the absence of legislation) render a just and equitable decision based on sound legal principles.

## Conclusion

Given the many advances in modern fertility therapies and the wide availability of these treatments, the notion of surrogacy finds itself on many social, family, religious and political agendas. Recognition of surrogacy and its important role in modern society is welcome, and today it no longer carries the stigma of an earlier time. Surrogacy is now optimistically viewed as being an acceptable method of procreation "in circumstances where it would otherwise not be possible". Irish courts dealing with a future issue where the surrogate fails to relinquish custody could apply the *Three Point Test *to consider genetic data and the intention of the parties at the time of making the contract. Account must also be taken of the best interests of the child, as determined by experienced judges.

For judicial matters, we favour the *Three Point Test *over existing expert committee opinions because there are important inconsistencies between documents Irish Courts might consider, including the CAHR Report [12] and the Brazier Report [34]. The CAHR Report found '*presumed*' intention to be critical in assigning legal parent status to the commissioning person/s, whereas the Brazier Report recommended the surrogate mother be recognised as the legal mother of the child irrespective of genetic relatedness or original parental intention.

On legislative matters, we agree with the CAHR Report's recommendation that the Oireachtas, in developing legislation on surrogacy, should consider the consensual nature of surrogacy arrangements, and, as such, the *'presumed intention' *of parenthood should be sufficient to grant the commissioning person/s legal parentage and custody of the child. The Oireachtas will also need to consider the unfavourable treatment of the commissioning person/s in the United Kingdom, where surrogacy contracts have been deemed unenforceable and would raise questions here about one's Constitutionally-protected right to procreate. This concern may be assuaged by application of the *Three Point Test *(or some similar methodology) as outlined here. For Ireland, this contract-based approach combined with a respect for Constitutional family rights may be regarded as a successful contribution to surrogacy arrangements here.

## Competing interests

The authors declare that they have no competing interests.

## Authors' contributions

ESS and CMH contributed equally to this manuscript.
